# Correlates of poor perinatal outcomes in non-hospital births in the context of weak health system: the Nigerian experience

**DOI:** 10.1186/1471-2393-14-341

**Published:** 2014-09-30

**Authors:** Peter Onubiwe Nkwo, Lucky Osaheni Lawani, Euzebus Chinonye Ezugwu, Chukwuemeka Anthony Iyoke, Agozie C Ubesie, Robinson Chukwudi Onoh

**Affiliations:** Department of Obstetrics & Gynecology, University of Nigeria Teaching Hospital Ituku-Ozalla Enugu Nigeria, Ozalla, Nigeria; Department of Obstetrics & Gynecology, Federal Teaching Hospital Abakaliki Nigeria, Abakaliki, Nigeria; Department of Pediatrics, University of Nigeria Teaching Hospital Ituku-Ozalla Enugu Nigeria, Ozalla, Nigeria

**Keywords:** Correlates, Perinatal mortality rate, Weak health system, Nigeria

## Abstract

**Background:**

Nigeria’s high perinatal mortality rate (PNMR) could be most effectively reduced by targeting factors that are associated with increased newborn deaths. Low access to skilled birth attendants (SBAs) and weak health system are recognized factors associated with high PNMR but other socio-demographic and reproductive factors could have significant influences as well. Identification of the major factors associated with high PNMR would be required in designing interventions to improve perinatal outcomes.

**Methods:**

For this cross-sectional study, data from the Nigeria Demographic and Health Survey 2008 were used to estimate the PNMR of non-hospital births in identified socio-demographic and reproductive situations that are known to influence PNMR. The estimated PNMR were compared using logistic regression analysis.

**Results:**

The PNMR was 36 per 1000 live births. North central region had the lowest PNMR while the south east region had the highest rate (odds ratio 1.59; 95% CI: 1.03, 2.45). Other correlates of high PNMR were belonging to the poorest wealth quintile (odds ratio 1.87; 95% CI: 1.30, 2.70), maternal age group 15–19 years (odds ratio 1.59; 95% CI: 1.05, 2.22), multiple birth (odds ratio 3.12; 95% CI: 2.11, 4.59), history of previous perinatal death (odds ratio 3.31; 95% CI: 2.73, 4.02), birth interval shorter than 18 months (odds ratio 1.65; 95% CI: 1.26, 2.17) and having a small birth size (odds ratio 2.56; 95% CI 1.79, 3.69). Birth attendant, place of birth, parity, maternal education and rural/urban residence had no association with PNMR.

**Conclusions:**

Reproductive factors that require midwifery skills were found to contribute most to PNMR. We recommend general strengthening of the health system, recruitment of SBAs and retraining of available birth attendants with emphasis on identification and referral of complicated cases. Family planning should be a core MCH activity to address the issues of teenage pregnancy and short pregnancy intervals.

## Background

It would be almost impossible to achieve the Millennium Development Goal 4 (MDG-4) at the current rate of decline of annual under-5 deaths from 10.5 million in 1990 to 6.6 million in 2012 [1–4]. The MDG-4 aims to reduce the under-5 mortality rate by two-thirds by the year 2015 using the 1990 rate as the baseline [5]. The least progress in meeting the MDG-4 target has been made with intrapartum-related newborn death which now contributes a higher proportion of the under-5 mortality than it did in 1990 [6, 7]. Intrapartum-related death alone was responsible for 2 million out of the 7.6 million yearly under-5 deaths reported in 2005 [1, 8] and comprises of fresh stillbirths and early neonatal deaths [8]. To meet the 2015 MDG-4 target, it would be necessary to identify and redress obstacles to satisfactory progress. Weak health systems and inadequate stock of skilled birth attendants (SBAs) as well as inequitable distribution of available SBAs have been identified as key challenges to achieving the MDG 4 & 5 targets [6, 7] especially in the high burden countries in sub-Saharan Africa and south Asia [9].

In Nigeria, there is an acute shortage of midwives and obstetricians, and a virtual unavailability of these cadres of health workers at the primary health care (PHC) level [10]. Moreover, due to poor condition of service, a large proportion of doctors and midwives migrate out of the country shortly after graduation [11, 12]. The available midwives are barely adequate to meet the needs of tertiary and city-based secondary hospitals [10]. In the meantime, non-midwife nurses and community health extension workers (CHEW) provide the bulk of maternity and newborn care services in Nigeria [10, 13]. The training curricula of both cadres do not meet the World Health Organization (WHO)/International Confederation of Midwives (ICM)/International Federation of Gynecology & Obstetrics (FIGO) minimum requirements for SBA [14, 15]. The over 70% of deliveries in some parts of Nigeria that takes place at home are usually attended by TBAs or family members [10, 16, 17]. Besides the state of health system and access to SBA, other socio-demographic, cultural and reproductive factors that influence pregnancy outcomes in each socio-political context also need to be identified and addressed in order to achieve optimal perinatal outcomes. These include the effects of economic status (wealth quintile), rural/urban residence [2, 18], maternal age [19], maternal education [20], parity [21], previous mortality experience [22], place of birth [23], multiple births [24], birth interval [25] and birth weight, among others.

With a population of over 140 million and perinatal mortality rate (PNMR) of 39 per 1000 live births, [16], Nigeria is the country with the largest newborn deaths in Africa and the second in the world [1–4]. The funding of the nation’s health system is partly responsible for the poor health indices. The health system is organized hierarchically into primary, secondary and tertiary levels [13, 26]. The federal government is responsible for funding tertiary health care, while the states and local government are responsible for secondary and primary health care respectively [13]. The primary level is the most basic care and is provided at the PHC centers [13]. Care provided at the PHC centers include the full range of basic maternal and child health care (MCH) services such as antenatal, delivery, postnatal and well-baby services among others [26]. Complicated pregnancies, labors and newborn illnesses that cannot be managed at the PHC centers are expected to be referred to the secondary level of care [13, 26]. The PHC centers are manned by nurses and CHEWs [13, 26]. It was originally planned that a functional PHC centre should be within five kilometer reach from any usual residential area [19]. This was to make healthcare accessible for the 70% of the population who live in the rural areas. The local government councils are responsible for the funding, staff recruitment and management of the PHC centers [13, 27]. Due to poor funding, only a few PHC centers are functional but these are without essential drugs and equipment, are poorly staffed and provide only day-time services as it requires a minimum of three nurses to provide 24 h services in a PHC facility [10, 26]. Moreover, there is neither formal referral system from the PHC clinics to the secondary level hospitals nor a formal transportation system to transport referred patients [10, 26]. Day-time service in Nigeria means that the clinics are open for eight hours (8 am-4 pm) from Mondays to Fridays. As many labors last longer that 8 h and many births occur at night and the weekends, most women are excluded from facility delivery even if they want to. Deliveries outside the health facilities are attended by the TBAs or elderly women in the family [17, 28].

Recent interventions by the federal government of Nigeria to improve pregnancy outcomes mainly target increasing the number of childbirths at the PHC clinics [26]. Media campaign encouraging women to have their childbirth at the PHC clinics is a major component of these interventions. These interventions do not include organized referral and transportation systems. It is unclear whether PHC facility births that are not supported by organized referral and transportation systems have better perinatal outcomes than home births in a weak health system with acute shortage of SBA, and whether increasing PHC facility births is the most cost-effective strategy to improve perinatal outcomes in Nigeria. A previous study based on the 2003 Nigeria DHS, failed to show that PNMR of PHC facility births was better than that of home births [29]. We believe that interventions intended to improve perinatal outcomes ought to be guided by knowledge of the major contributors to perinatal death in the target population. Such information would inform program planning and also provide baseline information that would be needed during program evaluation. The aim of the current study was to identify the factors that have significant associations with perinatal mortality in non-hospital childbirths in Nigeria. The specific objectives were to determine the PNMR in non-hospital births and to estimate the associations between perinatal mortality rate and some identified *a priori* socio-demographic and reproductive characteristics that are known to influence the PNMR. The result of the study is expected to provide evidence that would guide policies on perinatal health and also identify directions for further studies on perinatal health.

## Methods

### Study design and population

The study was based on an analysis of data from the Nigeria Demographic and Health Survey 2008 (Nigeria DHS 2008) which took place from June to October 2008 [16].The Nigeria DHS 2008 was a face-to-face nationally representative cross-sectional survey of women of reproductive age (15-49 yrs). Using the 2006 census enumeration area (EA) list as a sample frame, 888 (286 urban and 602 rural) EAs were selected from the 36 states and Federal Capital Territory (FCT) with each EA consisting of about 41 households. The target of the survey was to get 36,800 completed interviews. Based on the non-response rate of 2003 DHS, to achieve the sample size, 36, 800 households were selected and all age-eligible women were interviewed. Information was obtained from eligible respondents on a number of demographic and reproductive health issues including a detailed history of all children ever born alive, whether they were alive or dead at the time of interview and if dead, at what age they died. Information on place of birth and who assisted each birth was also obtained. They were also asked if they had ever had a previous pregnancy that did not result in live birth and how many months the pregnancy was when it terminated. The analysis for perinatal mortality in this study was based on the birth histories and on pregnancies that terminated at 28 weeks or older. The power for the survey was calculated to detect prevalence and effect estimates of key health indices at rural/urban residence, six regions and 36 states plus the FCT. It also has precision to detect differences in the estimates of the selected health indices including PNMR at the 5% level.

The main outcome measure for this study was the perinatal mortality rate. This was estimated from early neonatal deaths of births from 2003–2008; and stillbirths (pregnancies that lasted for 28 weeks or more but did not result in live birth) from 2003–2008. Early neonatal deaths and stillbirths were in turn respectively derived using the variables for year of birth ( b2) and age at death (b6 ) for early neonatal death, and year of non-viable pregnancy (v230 ) and its duration before it terminated ( v233) for stillbirth.

Birth attendant was the main exposure variable. Other *a priori* exposure variables that are known or thought to affect perinatal mortality include the following:

Demographic factors: region, residence (rural/urban), wealth index, mother’s age and mother’s education.

Reproductive factors: mother’s parity, previous mortality experience, place of delivery, number of babies (singleton or multiple) length of the birth intervals and size of baby at birth.

### Inclusion and exclusion criteria

Births at PHC centers, health posts, other non-hospital public and private places, respondents’ homes and other homes were included in the main analyses. Supplementary estimation of the PNMR of hospital births (births at government hospitals and private hospitals combined) was done for the purpose of comparison with the PNMR of non-hospital births where appropriate. We reported the PNMR of hospital births only in those circumstances where the pattern of perinatal death in hospital births was different from that of non-hospital births.

### Checks and management of the data

The dataset obtained online from Measure Evaluation^R^ was already cleaned and recoded. Missing dates were not allowed as dates were calculated and imputed for them. Missing values, inconsistent and impossible values and “I don’t know” responses were assigned special value. Such values were identified and recoded as missing values for purpose of the current analysis. In order to answer the research question, we generated a number of new variables from existing variables and recoded some variables.

### Statistical analysis

The data analysis was done with Stata^R^ statistical package version 12. Descriptive and logistic analyses were used to estimate and compare the PNMR across identified demographic and reproductive characteristics. Observed differences were considered significant at the p value of <0.05; 95% confidence interval. The “gen weight” and “svyset” command functions of the stata statistical software were used to account for the complex survey features of the HDS dataset.

### Ethical approval

The ethical considerations and approval for the collection of the primary data has been described [16]. Permission for the use of the data for this study was granted by Measure Evaluation®, the copyright holder of the dataset.

## Results

The overall response rate of the DHS 2008 was 94.9%. Out of a total of 32, 394 births, 25, 817 (79.7%) were non-hospital births while 6,577 (20.3%) were hospital births. The national perinatal mortality rate (PNMR) of non-hospital births in Nigeria was 36 per 1000 live births (95% CI: 33.4-39.3). This is lower than the PNMR of 46 per 1000 live births for hospital births (95% CI: 40–53.3).

### Descriptive analysis

#### Distribution of perinatal mortality of non-hospital deaths according to socio-demographic factors

The PNMR was lowest in the north central region (27/1000) and highest in the south east region (47/1000). The rate was 37/1000 in the rural areas and 35/1000 in the urban areas. Those in the richer wealth quintile had the lowest PNMR of 28/1000 while those in the richest quintiles had the highest rate (47/1000). However among hospital births, the PNMR showed a different pattern as follows: 76.6/1000 (95% CI: 5.453, 10.67) for poorest, 52.4/1000 (95% CI: 3.528, 7.704) for poorer, 55.2/1000 (95% CI: 4.174, 7.268) for middle, 44.2/1000 (95% CI: 3.436, 5.781) for richer, and 40.2/1000 (95% CI: 3.112, 5.188) for the richest, p = 0.007. Maternal age group 25–29 years was associated with the lowest PNMR (33/1000) while the age group 15–19 years was associated with the highest rate (50/1000). The PNMR increased with increasing level of maternal formal education with higher education being associated with the highest rate. For hospital births, the PNMR showed an inverse relationship with the level of mother’s education thus: 64/1000, 53/1000, 41.4/1000 and 38/1000 respectively for no maternal education, primary education, secondary education and tertiary education (P = 0.0736). The distribution of perinatal mortality in non-hospital births according to socio-demographic factors is shown in Table 1.

**Table 1:** **Distribution of perinatal mortality of non-hospital births in Nigeria according to socio-demographic factors (2003–2008) (N** ^**1**^ **= 25, 817)**

Demographic characteristics	Number ^2^	PNMR ^3^ (95% CI ^4^ )	P value
**Region**			
**North central**	109	30 (26.7-38.0)	0.0257
**North east**	269	39 (35.3-46.6)	
**North west**	301	36 (33.6-43.3)	
**South east**	72	47 (39.1-59.7)	
**South south**	111	42 (35.2-52.4)	
**South west**	55	29 (25.9-43.3)	
**Residence**			
**Urban**	169	35 (28.9-42.0	
**Rural**	748	37 (33.5-40.1)	0.6406
**Wealth index**			
**poorest**	328	41 (35.7-46.8)	0.0117
**Poorer**	259	36 (31.2-42.5)	
**Middle**	157	31 (25.7-37.2)	
**Richer**	102	28 (22.3-36)	
**Richest**	71	47 (35.2-62.1)	
**Age group**			
**15-19**	71	50 (38.8-65.2)	0.1605
**20-24**	190	38 (32.2-45)	
**25-29**	225	33 (28.2-38.2)	
**30-34**	182	34 (28.6-40.8)	
**35-39**	127	34 (27.2-41.7)	
**40-44**	83	41 (30.9-54.3)	
**45-49**	39	42 (28.6-61.7)	
**Mother education**			
**No education**	541	35 (31.9-39.3)	0.7301
**Primary**	205	36 (29.9-42.1)	
**Secondary**	153	39 (32.5-47.5)	
**Higher**	18	43 (24.6-72.5)	

^1^Total number of births.

^2^Number of perinatal deaths.

^3^Perinatal mortality per 1000 live births.

^4^Confidence interval.

#### Distribution of perinatal mortality of non-hospital births according to reproductive factors

With a PNMR of 33/1000, parity 2–4 was associated with the lowest perinatal deaths while primiparity was associated with the highest rate (39/1000). Those with a history of previous mortality experience were at a much higher risk of perinatal death than those without such history (79/1000 versus 28/1000). Delivery at private frontline health facility was associated with lower perinatal deaths than delivery at public PHC facility. The perinatal mortality associated with multiple births was 121/1000 compared to 34/1000 for singleton births. At a PNMR of 28/1000, birth interval of 25 months and above was associated with much lower perinatal deaths than the shorter birth intervals while an interval of less than 18 months was associated with the highest perinatal mortality. Births assisted by CHEWs were associated with a PNMR of 31/1000 compared to 41/1000 for those assisted by nurses. Being born small was associated with a PNMR of 75/1000 while having “average” birth size was associated with a PNMR of 30/1000. Table 2 shows the distribution of perinatal mortality according to reproductive factors.

**Table 2:** **Distribution of perinatal mortality of non-hospital births in Nigeria according to selected reproductive factors (2003–2008) (N** ^**1**^ **= 25, 817)**

Reproductive factors	Number ^2^	(95% CI ^3^ )	P value
**Parity group**			
**Para 1**	80	39 (31.2-49.2)	0.1227
**Para 2-4**	385	33 (29.4-37.5)	
**Para ≥5**	452	39 (34.5-43.4)	
**Previous mortality experience**			
**Yes**	330	79 (70.0-89.5)	
**No**	587	28 (25.4-30.5)	<0.001
**Place of delivery**			
**Hospitals** ^4^	306	46 (40–53.3)	
**Woman's home**	591	35 (31.5-38.3)	0.046^5^
**Other home**	63	37 (27.4-49.2)	
**Govt health centre**	69	35 (26.5-47.3)	
**Govt health post**	6	42 (17.5-99.4)	
**Other public**	5	78 (29.3-191.7)	
**Other private**	3	24 (7.4-74.4)	
**Others**	14	31 (14.9-65.3)	
**Multiple birth**			
**Multiple**	96	121 (93.1-155)	<0.001
**Singleton**	821	34 (30.9-36.6)	
**Birth interval**			
**<18 months**	170	73 (61.8-86.9)	<0.001
**18-24 months**	139	33 (27.7-39.2)	
**≥ 25 months**	406	28 (25.2-31.5)	
**First birth**	202	48 (40.8-55.5)	
**Birth assistant**			
**Nurse**	99	41 (32.2-51.5)	0.7932
**CHEW**	23	31 (17.0-54.6)	
**TBA**	231	35 (30.3-41.0)	
**Family & friends**	192	33 (28.2-39.0)	
**Others**	18	35 (20.6-59.4)	
**No one**	178	34 (28.4-40.0)	
**Size at birth**			
**Big**	124	33 (27.1-40.4)	<0.001
**Above average**	173	31 (27.2-37.3)	
**Average**	259	28 (28.7-37.2)	
**Below average**	91	41 (38.0-58.7)	
**Small**	86	75 (58.7-94.4)	

^1^Total number of births.

^2^Number of perinatal deaths.

^3^Confidence interval.

^4^Information on hospital births were added in this table for the purpose of comparison only.

^5^This p value applies only when hospital births are compared with home births.

### Regression analysis and estimates of crude odds ratio (OR) and adjusted odds ratio (AOR) of perinatal death of non-hospital births according to socio-demographic and reproductive factors

Table 3 shows the crude odds ratios (OR) and adjusted odds ratios (AOR) of perinatal death associated with selected socio-demographic and reproductive factors. Using perinatal death as the primary outcome and birth attendant as the main exposure variable, the factors that retained significant influence on perinatal mortality after adjusting for potential confounding factors included living in the south east region (AOR = 1.59; 95% CI: 1.03, 2.45 p = 0.034), belonging to the poorest economic status (AOR = 1.87; 95% CI: 1.3, 2.7 p = 0.001), age ≤19 yr (AOR = 1.52; 95% CI: 1.05, 2.22 p = 0.028), previous mortality experience (AOR = 3.31; 95% CI: 2.73, 4.02 p < 0.001), multiple gestation (AOR = 3.12; 95% CI: 2.11, 4.59 p < 0.001), birth interval ≤18 months (AOR = 1.65; 95% CI: 1.26, 2.17 p ≤ 0.001) and small birth size (AOR = 2.57; 95% CI: 1.79, 3.69 p < 0.001).

**Table 3:** **Odds ratio of perinatal death in non-hospital births in Nigeria according to birth attendant adjusted for** ***a priori*** **confounding factors (2003–2008) (N** ^**1**^ **= 25, 817)**

Demographic and reproductive characteristics	Crude OR	AOR ^2^ (95% CI ^3^ )	P value
**Birth attendant**			
**CHEW**	1(base)	1(base)	
**Nurse**	1.34	1.42 (0.75-2.67)	0.278
**TBA**	1.16	0.98 (0.53-1.80)	0.943
**Family/friends**	1.08	0.93 (0.49-1.75)	0.817
**Others**	1.15	0.93 (0.43-1.04)	0.865
**No assistance**	1.10	0.90 (0.47-1.72)	0.752
**Region**			
**North central**	1(base)	1(base)	
**North east**	1.47	`1.29 (0.96-1.72)	0.087
**North west**	1.35	1.29 (0.967-1.73)	0.083
**South east**	1.78	1.59 (1.03-2.45)	0.037
**South south**	1.57	1.49 (0.972.27)	0.066
**South west**	1.06	1.07 (0.66-1.75)	0.782
**Residence**			
**Urban**	1(base)	1(base)	
**Rural**	1.05	1.05 (0.82-1.35)	0.676
**Wealth index**			
**Richer**	1	1(base)	
**poorest**	1.46	1.87 (1.30-2.70)	0.001
**Poorer**	1.30	1.69 (1.19-2.41)	0.004
**Middle**	1.09	1.30 (0.92-1.84)	0.144
**Richest**	1.69	1.54 (0.99-2.37)	0.053
**Mother’s age**			
**25-29**	1(base)	1(base)	
**15-19**	1.56	1,52 (1.05-2.22)	0.028
**20-24**	1.16	1.24 (0.95-1.62)	0.107
**30-34**	1.04	1.08 (0.821.41)	0.589
**35-39**	1.03	0.90 (0.66-1.24)	0.523
**40-44**	1.26	1.19 (0.79-1.80)	0.407
**45-49**	1.29	1.15 (0.67-1.96)	0.615
**Mother’s education**			
**No education**	1(base)	1(base)	
**Primary**	1	1.05 (0.82-1.34)	0.692
**Secondary**	1.11	0.99 (0.71-1.38)	0.949
**Higher**	1.21	0.87 (0.39-1.94)	0.727
**Parity**			
**Para2-4**	1(base)	1(base)	
**Para1**	1.90	0.69 (0.47-1.01)	0.058
**Para ≥5**	1.17	1.06 (0.81-1.37)	0.678
**Previous mortality experience**			
**No**	1(base)	1(base)	
**Yes**	3.01	3.31 (2.73-4.02)	<0.001
**Place of birth**			
**Other private**	1(base)	1(base)	
**Respondent home**	1.47	2.63 (0.59-11.79)	0.206
**Other home**	1.56	2.28 (0.50-10.36)	0.288
**Govt PHC centre**	1.50	2.25 (0.50-10.02)	0.286
**Govt health post**	1.81	5.27 (0.94-29.44)	0.058
**Multiple birth**			
**No**	1(base)	1(base)	
**Yes**	3.95	3.12 (2.11-4.59)	<0.001
**Birth interval**			
**≥25 months**	1(base)	1(base)	
**<18 months**	2.73	1.65 (1.26-2.17)	<0.001
**18-24 months**	1.18	1.20 (0.96-1.52)	0.113
**First births**	1.73	2.22 (1.64-3.01)	<0.001
**Birth size**			
**Average**	1(base)	1(base)	
**Above average**	1.04	1.03 (0.79-1.33)	0.854
**Big**	1.10	1.16 (0.87-1.56)	0.317
**Below average**	1.37	1.26 (0.92-1.72)	0.150
**Small**	2.61	2.55 (1.79-3.69)	<0.001

^1^Total number of births.

^2^Adjusted odds ratio: The adjusted odds ratios reported here were derived from the multivariable regression analysis that contained all the demographic and reproductive variables in Tables 1 and 2 in a single regression model.

^3^Confidence interval.

## Discussions

The main findings of the study were:

1. The PNMR among non-hospital births in Nigeria (2003–2008) was 36 per 1000 live births while the PNMR of hospital births was 46 per 1000 live births.
2. After adjusting for known confounding factors using multiple regression analysis, there was no significant difference in perinatal mortality according to rural versus urban residence, birth assistant and place of birth. Reproductive characteristics such as teenage pregnancy, short birth interval, multiple gestation, bad obstetric history and small birth size were found to be the major contributors to the PNMR among non-hospital births in Nigeria.

### Perinatal mortality rate of non-hospital births

The PNMR was 36/1000 live births in births that occurred at the primary health care facilities and homes. This was lower than the PNMR of 46/1000 live births when only hospital births were analyzed and also lower than the PNMR of 39/1000 live births when hospital births and non-hospital births were combined [16] (result not included), and much lower than the PNMR in a previous hospital-based study [30].The higher mortality rates of hospital-based studies are believed to result from referral bias [31] where serious and terminal cases are referred to hospitals. The higher PNMR associated with the receiving facilities in the referral cycle is mostly attributable to delays at home or PHC clinic in deciding to seek care at hospital, delays in transportation from home or PHC clinic to hospital and delays in accessing the appropriate care when the case has reached the receiving hospital. Whereas referral of complicated labors from homes to PHC clinics was a possibility in the current study, however in Nigeria the usual flow of referral is from homes to hospitals and from PHC clinics to hospitals. This is due to a general belief that complicated labors might require surgical interventions or the use of some equipment that are not usually available at the PHC clinics. The non-significant difference in the PNMR between PHC facility births and home births also suggests that referral of complicated home labors to the PHC clinics might not have occurred at a large scale.

### Distribution of perinatal mortality of non-hospital births according to socio-demographic factors

The PNMR was highest in the south east region (47/1000) and lowest in north central region (27/1000). The relatively higher PNMR in the south east region was a departure from previous DHS patterns where the region shared the best reproductive and child health indicators with the south west region [32]. The result of a recently concluded DHS is eagerly awaited to show whether the DHS 2008 finding was an isolated case or whether a new PNMR pattern has emerged^a^.

Perinatal mortality was higher in the rural than the urban areas (37/1000 versus 35/1000). This is consistent with previous Nigeria DHS surveys and the global pattern [2, 32, 33]. However, the odds ratio of 1.05 (95% CI: 0.85-1.31; p = 0.641) suggests that the evidence for a true difference was weak. There are probably stronger common risk factors for perinatal death among women who choose to deliver at the PHC centers or at home than the rural/urban influence.

People in the richer and middle wealth quintiles had lower PNMR than the poorer and the poorest. It is noteworthy that the very high PNMR initially observed in association with the richest wealth status on descriptive analysis disappeared following multiple adjustment for confounding factors suggesting that some factors other than high economic status were responsible for the observed association. Note also that for hospital births, the PNMR was consistently highest in the poorest socio-economic status and lowest in the richest wealth group. Because government-owned hospitals and private hospitals in Nigeria charge service fees, the rich are more likely than the poor to choose hospitals for prenatal and perinatal care. On the other hand, a larger proportion of the poor than the rich who reported hospital birth might have been referred to hospital from homes and from PHC clinics as complicated cases which have poorer prognosis.

As in other studies [19, 28], extremes of maternal age was found to be associated with increased PNMR. But contrary to most previous studies [20], mother’s education was not found to be associated with improved perinatal outcomes in non-hospital births. This suggests that among those who chose to deliver at; or are forced to deliver at the PHC facilities or homes, other residual factors exert greater influence on perinatal mortality than maternal education. Moreover, the effect of mother’s education on perinatal mortality acts mostly through the pathway of choice and uptake of health services [34]. Childbirth at a suboptimal environment would not be the best choice expected of an educated woman. Note the inverse relationship between PNMR and mother’s level of education in hospital births. The relationship between PNMR and mother’s education observed among the hospital births is consistent with previous studies [20] and also supports the opinion that the positive influence of education on perinatal outcomes operates through the pathway of choice of services [34]. All things being equal, an educated mother is more likely than her less educated counterpart to choose hospital instead of home or PHC clinic for childbirth. Furthermore, some Nigerian cultures forbid talking about dead children and miscarriages [16]. Proportionately more among the less educated women were likely to observe these cultural practices and hence report fewer than their true perinatal deaths. It needs to be appreciated also that in a situation of very weak health system such as Nigeria, the positive influence of mother’s education on child survival would likely become increasingly greater after the perinatal period when the home environment plays comparatively greater role in child survival than the circumstances of childbirth.

### Distribution of perinatal death of non-hospital births according to reproductive factors

Parity group 2–4 was found to be associated with the lowest PNMR. Other studies have also identified primiparity and grand-multiparity as risk factors for perinatal death [21, 35]. Comparison of home births with PHC facility births did not show significant difference in PNMR. This is the pattern of perinatal mortality in well-organized and richly-resourced health systems where home births are planned and assisted by skilled birth attendants [23]. In such systems only low risk pregnancies qualify for home birth and there are also functional ambulance services and effective linkage with functional hospitals in the events of emergencies. The situation in Nigeria is quite different as it appears that risk assessment was not a criterion for choosing home versus facility birth. The analysis of place of delivery by previous mortality experience revealed that the great majority of those with history of previous perinatal deaths still chose home birth after such experiences (result not shown). Therefore in a situation where high risk pregnancies undergo home births that are neither assisted by skilled birth attendants nor have access to emergency obstetric and newborn care services, much higher PNMR was expected from home births than PHC clinic births provided that the PHC clinics were providing better quality care for similar cases than the homes. On the other hand, if mismanaged home labors were being referred to the PHC centers, the PNMR at the PHC clinics would then be expected to be higher than that of home births (see the high PNMR of hospital births which was attributable to referral bias). Referrals of complicated labors from homes to the PHC facilities were not likely to be significant as the PHC clinics provided only day-time services and were not designed to provide comprehensive emergency obstetric services. Referrals were more likely to be from homes to the hospitals rather than to PHC clinics.

Multiple birth was found to be associated with over threefold increase in perinatal mortality compared with singleton births (AOR = 3.9; 9% CI 2.93-5.32; p < 0.001). The perinatal outcome of multiple pregnancies is dependent on the quality of care during pregnancy, childbirth and postpartum period. With standard quality of care, PNMR of twin births do not differ significantly from singleton births [36]. Unlike well-resourced health systems, there is increased risk of perinatal death among the twins and higher order births compared to singleton births in settings with poor quality maternity services [24]. Multiple pregnancies and multiple births are cases for hospital management by skilled midwives and obstetricians, not for PHC facility or home delivery by unskilled birth attendants. Hence in this study, the PNMR of 121/1000 in multiple births was much higher than 34/1000 in singleton births partly because of the weak health system and poor skills of the birth attendants.

Birth interval of greater than two years was found to be associated with the best perinatal outcome; those born less than 18 months after a previous birth fared the worst (AOR = 2.73; 95% CI 2.25-3.31; p < 0.001). Short birth interval is a recognized risk factor for perinatal death [25]. This finding draws attention to the importance of family planning as a core strategy for achieving the MDG 4 & 5 goals. Family planning services are currently not available at most PHC clinics in Nigeria.

Perinatal mortality rate in birth assisted by the CHEWs was 31/1000 compared with 41/1000 for those assisted by nurses (Table 2). The associated adjusted odds ratio of 1.34 (95% CI, 0.70-2.57, p = 0.374) suggests that there was no significant difference between these two categories of birth attendants. It is important to note that respondents in the DHS surveys are likely to misclassify CHEW and nurses as both cadres perform similar functions and wear similar uniforms at the PHC clinics. Moreover, “nurse” is the generic job title for female health workers in most rural communities in Nigeria. Therefore more CHEWs were probably misclassified as nurses than vice versa. Compared with the CHEW, the odds ratios for perinatal death in births assisted by the other categories of birth attendants were 1.16 for TBA, 1.08 for family/friend, 1.1 for others and 1.1 for no assistance at all. The CHEW thus appeared to be the safest frontline birth assistant at the PHC facilities and communities. A search of electronic databases including Medline, EMBASE, Scopus, Reproductive Health Library and CINAHL Plus did not find any prior study that compared perinatal outcomes of births assisted by CHEWs and nurses.

Average birth size was associated with the lowest PNMR while small birth size had the greatest risk of perinatal death. Low birth weight as a result of prematurity or intrauterine growth restriction is a recognized risk factor for perinatal death [37].

In summary, the findings of this study suggest that women who give birth at home or at PHC facilities in Nigeria have some common underlying residual socio-demographic risk factors for poor perinatal outcomes that overshadow the positive influences of wealth and education. Cultural practices including religious practices could exert such a homogenizing influence. Further studies are required to uncover such factors. On the other hand, the *a priori* adverse influences of the reproductive factors on perinatal mortality were exaggerated. The negative effects of such reproductive factors as multiple births and previous mortality experiences could be effectively minimized by early identification and referral of such cases to hospitals as the PHC clinics are disallowed to manage complicated pregnancies such as these. Teenage pregnancies and short pregnancy intervals are best prevented through appropriate family planning interventions. Unfortunately, the weak health system and poor clinical skills (including poor referral skills) of birth attendants permitted these factors to exert their maximum negative influences on perinatal mortality. When the high PNMR (36/1000) of non-hospital births is considered together with the even higher PNMR (46/1000) of hospital births, the picture becomes clearer that of a health system that is too weak to respond to the basic demands of safe perinatal care. Many of the referred cases reach the hospitals too late to be rescued and some of the hospitals are not adequately equipped to manage the referred cases.

## Conclusions

The factors that contribute most to perinatal death in non-hospital births in Nigeria include teenage pregnancy, short pregnancy interval, multiple gestations, previous perinatal deaths and small size at birth. The myriads of adverse pregnancy outcomes resulting from teenage pregnancy and short pregnancy interval are effectively preventable by family planning. Multiple gestations and the history of previous perinatal deaths are cases for hospital care that should not be managed at homes or at the PHC clinics. The absence of improved PNMR in PHC facility births when compared with home births could be due to lack of SBAs at the PHC clinics, referral bias or because high-risk pregnant women preferentially selected to have childbirth at PHC facilities rather than at home. However, the consistently high PNMR at all locations of birth including hospitals suggests a health system that is generally weak.

We recommend general strengthening of the health system including provision of life-saving equipment, medicines and consumables, recruitment of trained SBAs and adequate competency-based retraining of existing birth attendants as well as establishment of functional referral and transportation systems before mobilizing the public for increased uptake of PHC facility-based perinatal services. The emphases of SBA training and retraining should include the skills to identify complicated pregnancies and labors that must be referred to hospital without delay. Referral hospitals should be equipped with requisite life-saving equipment and skilled personnel to competently manage complicated cases referred from homes and PHC clinics. We further recommend that family planning should henceforth be made one of the core MCH activities because of its numerous benefits for perinatal health. Finally, public awareness should be created on the benefits of family planning and on the dangers of managing high-risk pregnancies and labors at home or at the PHC clinics. We believe that these recommendations would also benefit other countries with similar weak health systems as Nigeria.

### Strengths and limitations of the study

#### Strengths

Nigeria DHS 2008 was a nationally representative survey with large sample size and associated high power and precision to detect differences in the indicators of interest including perinatal mortality. The response rate of 94.9% was high for such a large population survey. To ensure accuracy and uniformity of information collected, the study questionnaire was translated to local languages and back-translated to English, contained consistency check questions, and were administered by trained field officers with multiple levels of supervision. Missing values were minimal. The study was therefore based on robust data base. Although the DHS 2008 was a cross-sectional survey, the periodic repeats of the DHS have made it useful for tracking progress in indicators of interest.

### Limitations

The study was based on respondents’ recall of previous events which is prone to recall bias. The error in remembering the age at death involving very young ages is a recognized problem in DHS and other surveys [16]. Moreover, perinatal death is an emotional event which some women would prefer not to remember. In addition some cultures in Nigeria forbid talking about previous perinatal deaths [16]. This could have led to underestimation of the true magnitude of perinatal deaths especially among the less educated respondents. The calculation of perinatal death using the DHS dataset has recognized challenges including the difficulty of determining stillbirths; and calculation of intrapartum-related stillbirth is probably impossible with the DHS dataset.

It is intuitive that women with higher-risk pregnancies are more likely than their lower-risk counterparts to have health facility childbirths by choice or through referral. This could have lead to worse perinatal outcomes in the PHC facility births compared to home births. The DHS dataset did not contain the information needed to adequately control for these potential risk-related biases.

There were probably some misclassification of birth assistants by the respondents as the midwives, nurses and CHEWs wear similar uniforms and perform similar duties at the PHC facilities. Because the generic job title of female health workers in most rural communities in Nigeria is “nurse”, some CHEWs might have been misclassified as nurses. Misclassification in the reverse direction is possible but less likely.

These limitations are acknowledged.

## Endnote

^a^The 2013 Nigeria DHS showed that the south east region had the second lowest PNMR at 36/1000.

## References

[1] World Health Organization Organization (2005). World Health Report 2005: Make Every Mother and Child Count. WHO.

[2] Black RE, Morris SS, Bryce J (2003). Where and why are 10 million children dying every year?. Lancet.

[3] Bhutta ZA, Chopra M, Axelson H, Berman P, Boerma T, Bryce J, Bustreo F, Cavagnero E, Cometto G, Daelmans B, de Francisco A, Fogstad H, Gupta N, Laski L, Lawn J, Maliqi B, Mason E, Pitt C, Requejo J, Starrs A, Victora CG, Wardlaw T (2010). Countdown to 2015 decade report (2000–10): taking stock of maternal, newborn, and child survival. Lancet.

[4] World Health Organization (2013). Estimation method for child mortality Used in: Level and Trends of Child mortality - Report 2013 Estimates developed by the WHO, UNICEF, UN Population Division.

[5] United nations General Assembly (2001). Report of the Secretary-General. Road map towards the implementation of the United Nations Millennium Declaration.

[6] Lawn JE, Lee AC, Kinney M, Sibley L, Carlo WA, Paul VK, Pattinson R, Darmstadt GL (2009). Two million intrapartum-related stillbirths and neonatal deaths: where, why, and what can be done?. Int J Gynecol Obstet.

[7] Lawn JE, Kinney M, Lee AC, Chopra M, Donnay F, Paul VK, Bhutta ZA, Bateman M, Darmstadt GL (2009). Reducing intrapartum-related deaths and disability: Can the health system deliver?. Int J Gynecol Obstet.

[8] Lawn J, Shibuya K, Stein C (2005). No cry at birth: Global estimates of intrapartum stillbirths and intrapartum-related neonatal deaths. Bull World Health Organ.

[9] Bhutta ZA, Lassi ZS, Mansoor N (2010). Systematic Review on Human Resources for Health Interventions to Improve Maternal Health Outcomes: Evidence from Developing Countries: World health organization (WHO).

[10] Federal Ministry of Health (2008). National Human Resources for Health Strategic Plan (2008–2012).

[11] Ihekweazu C, Anya I, Anosike E (2005). Nigerian medical graduates: Where are they now?. Lancet.

[12] Eastwood JB, Conroy RE, Naicker S, West PA, Tutt RC, Plange-Rhule J (2005). Loss of health professionals from sub-Saharan Africa: The pivotal role of the UK. Lancet.

[13] Abdulraheem IS, Olapipo AR, Amodu MO (2012). Primary health care services in Nigeria: Critical issues and strategies for enhancing the use by the rural communities. J Public Health Epidemiol.

[14] International Confederation of Midwives: **Essential Competencies for Basic Midwifry Practice 2010, ICM 2013.** Available at http://www.internationalmidwives.org/assets/uploads/documents/CoreDocuments/ICM%20Essential%20Competencies%20for%20Basic%20Midwifery%20Practice%202010,%20revised%202013.pdf. Accessed on February 26, 2014

[15] Adegoke AA, Mani S, Abubakar A, Van Den Broek N (2013). Capacity building of skilled birth attendants: A review of pre-service education curricula. Midwifery.

[16] National Population Commission (NPC) [Nigeria] and ICF Macro (2009). Nigeria Demographic and Health Survey 2008.

[17] Galadanci HS, Ejembi CL, Iliyasu Z, Alagh B, Umar US (2007). Maternal health in Northern Nigeria - A far cry from ideal. BJOG.

[18] Victora CG, Wagstaff A, Schellenberg JA, Gwatkin D, Claeson M, Habicht JP (2003). Applying an equity lens to child health and mortality: More of the same is not enough. Lancet.

[19] Kumar C, Singh PK, Rai RK, Singh L (2013). Early neonatal mortality in India, 1990–2006.[Erratum appears in J Community Health. 2013;38(1):131–2]. J Commun Health.

[20] Kraft AD, Nguyen KH, Jimenez-Soto E, Hodge A (2013). Stagnant neonatal mortality and persistent health inequality in middle-income countries: a case study of the Philippines. PLoS ONE [Electronic Resource].

[21] Kalter HD, Khazen RR, Barghouthi M, Odeh M (2008). Prospective community-based cluster census and case–control study of stillbirths and neonatal deaths in the West Bank and Gaza Strip. Paediatr Perinat Epidemiol.

[22] Malik SJ, Mir NA (1992). Perinatal mortality in high risk pregnancy: a prospective study of preventable factors. Asia Oceania J Obstet Gynaecol.

[23] de Jonge A, van der Goes BY, Ravelli AC, Amelink-Verburg MP, Mol BW, Nijhuis JG, Bennebroek Gravenhorst J, Buitendijk SE (2009). Perinatal mortality and morbidity in a nationwide cohort of 529 688 low-risk planned home and hospital births. BJOG.

[24] Olusanya BO (2011). Perinatal outcomes of multiple births in southwest Nigeria. J Health Popul Nutr.

[25] Cecatti JG, Correa-Silva EPB, Milanez H, Morais SS, Souza JP (2008). The associations between inter-pregnancy interval and maternal and neonatal outcomes in Brazil. Matern Child Health J.

[26] Abimbola S, Okoli U, Olubajo O, Abdullahi MJ, Pate MA (2012). The midwives service scheme in Nigeria. PLoS Medicine.

[27] Asuzu M (2005). Commentary: The necessity for a health systems reform in Nigeria. J Community Med Prim Health Care.

[28] Chukwuani CM, Olugboji A, Akuto EE, Odebunmi A, Ezeilo E, Ugbene E (2006). A baseline survey of the primary healthcare system in south eastern Nigeria. Health Policy.

[29] Oji OS (2009). Risk factors for perinatal mortality in Nigeria: the role of place of delivery and delivery assistants. Thesis.

[30] Kuti O, Orji E, Ogunlola I (2003). Analysis of perinatal mortality in a Nigerian teaching hospital. J Obstet Gynecol.

[31] Tüchsen F, Andersen O, Olsen J (1996). Referral bias among health workers in studies using hospitalization as a proxy measure of the underlying incidence rate. J Clin Epidemiol.

[32] National Population Commission (NPC) Nigeria and ICF Macro (2004). Nigeria Demographic and Health Survey 2003.

[33] Unicef WHO (2004). Why are 4 million newborn babies dying each year?. Lancet.

[34] Faundes A, Hardy E, Diaz J, Pinotti J (1982). Association of marital status and years of schooling with perinatal outcome: The influence of pre-natal care as an intermediate variable. J Perinat Med.

[35] Ntambue AM, Donnen P, Dramaix-Wilmet M, Malonga FK (2012). [Risk factors for perinatal mortality in the city of Lubumbashi, Democratic Republic of Congo]. Revue d Epidemiologie et de Sante Publique.

[36] Ward Platt MP, Glinianaia SV, Rankin J, Wright C, Renwick M (2006). The North of England Multiple Pregnancy Register: Five-year results of data collection. Twin Res Hum Genet.

[37] Moraitis AA, Wood AM, Fleming M, Smith GC (2014). Birth weight percentile and the risk of term perinatal death. Obstet Gynecol.

[CR38] The pre-publication history for this paper can be accessed here: http://www.biomedcentral.com/1471-2393/14/341/prepub

